# Dairy products and the risk of developing prostate cancer: A large‐scale cohort study (JACC Study) in Japan

**DOI:** 10.1002/cam4.4233

**Published:** 2021-10-04

**Authors:** Kazuya Mikami, Kotaro Ozasa, Tsuneharu Miki, Yoshiyuki Watanabe, Mitsuru Mori, Tatsuhiko Kubo, Koji Suzuki, Kenji Wakai, Masahiro Nakao, Akiko Tamakoshi, for the JACC Study Group

**Affiliations:** ^1^ Department of Urology, Graduate School of Medical Science Kyoto Prefectural University of Medicine Kyoto Japan; ^2^ Department of Urology Japanese Red Cross Kyoto Daiichi Hospital Kyoto Japan; ^3^ Department of Epidemiology for Community Health and Medicine, Graduate School of Medical Science Kyoto Prefectural University of Medicine Kyoto Japan; ^4^ Department of Epidemiology Radiation Effects Research Foundation Hiroshima Japan; ^5^ Department of Public Health Sapporo Medical University School of Medicine Sapporo Japan; ^6^ Department of Public Health and Health Policy, Graduate School of Biomedical and Health Sciences Hiroshima University Hiroshima Japan; ^7^ Department of Preventive Medical Sciences Fujita Health University School of Medical Sciences Toyoake Japan; ^8^ Department of Preventive Medicine/ Biostatistics and Medical Decision Making Nagoya University Graduate School of Medicine Nagoya Japan; ^9^ Department of Public Health Hokkaido University Graduate School of Medicine Sapporo Japan

**Keywords:** cohort study, dairy products, diet, epidemiology, prostate cancer

## Abstract

Dairy products have been indicated as a risk factor for prostate cancer. However, only a few epidemiological studies have reported dairy products as being a risk factor for prostate cancer in Japan, reporting contradictory results. We therefore investigated the association between the intake of dairy products and the occurrence of prostate cancer through a large‐scale cohort study. The Japan Collaborative Cohort study analyzed approximately 110,000 residents from various Japanese districts who participated in our questionnaire survey during 1988–1990. The subjects of the present study were 26,464 men (age range: 40–79 years) from 24 districts wherein cancer incidence was reported. Their clinical course was followed up until 2009. Hazard ratios (HRs) were calculated using Cox's proportional hazards model, adjusted for age, survey area, family history of prostate cancer, body mass index, and total energy intake. For diet, we calculated the HRs associated with intermediate and high consumption of dairy products and compared them with those associated with low consumption. There were 412 cases of prostate cancer in the survey population. As dairy products, milk, yogurt, cheese, and butter were evaluated. Among them, milk consumption was associated with a significant risk (HR = 1.37, *p* = 0.009) and a dose‐dependent response (*p* for trend = 0.009) adjusted for age and family history of prostate cancer, stratified by area. Milk and yogurt consumption showed a significantly positive risk and a dose–response relationship adjusted for age, family history of prostate cancer, body mass index, and total energy intake, stratified by area. In summary, a high intake of dairy products such as milk increased the risk of developing prostate cancer in Japanese men.

## Graphical abstract

This study investigates the association between consumption of dairy product and development of prostate cancer using a large‐scale cohort study in Japan. Our study makes a significant contribution to the literature because we were able to show statistically significant associations and dose–response relationships between high intake of milk and yogurt and prostate cancer, even after adjusting for potential confounders including body mass index and total energy intake.

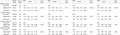

## 1 INTRODUCTION

Although the incidence of prostate cancer in Western countries has always been high, this was in contrast to that in Japan in the past. However, the incidence of prostate cancer in Japan has increased rapidly. Recently, prostate cancer has become one of the most common cancers in men. While many epidemiological studies, including cohort studies, have conducted investigations on this cancer in the Western world, only relatively few studies have been reported from Japan.^1^, ^2^, ^3^, ^4^, ^5^, ^6^, ^7^

Prostate cancer is one of the androgen‐dependent cancers. Age, family history of prostate cancer, and race are well‐known risk factors. In addition, total energy intake and obesity have been reported as risk factors. Intake of dairy products has also been indicated as a risk factor.^8^, ^9^, ^10^ Although several case–control and cohort studies have reported a positive correlation between the occurrence of prostate cancer and dairy product consumption, the results have been contradictory. In approximately 50% of these studies, the intake of dairy products was found to be a significant risk factor.^8^, ^9^, ^10^ Recent meta‐analyses showed positive associations between dairy product consumption and prostate cancer development,^11^, ^12^ and many studies showed significant relationships between the intake of dairy products and occurrence of advanced prostate cancer.^13^, ^14^

In Japan, dairy products are not part of the traditional diet. However, the intake of dairy products has increased recently. According to The National Health and Nutrition Survey in Japan, intake of dairy products per day for each person increased rapidly from 103.5 g in 1975 to 122.2 g in 1988 and increased gradually to 125.1 g in 2009.^15^ Several decades ago, some Japanese studies investigated the association between the intake of dairy products and occurrence of prostate cancer.^1^, ^2^ A case–control study reported a positive but nonsignificant risk associated with milk consumption through a semi‐quantitative food frequency questionnaire.^6^ A cohort study showed a significant positive risk associated with dairy product consumption and a dose–response relationship.^7^ Therefore, we investigated the relationship between the risk of developing prostate cancer and consuming dairy products in a large‐scale cohort study in Japan.

## 2 SUBJECTS AND METHODS

The Japan Collaborative Cohort (JACC) study was conducted based on a subsidy for scientific research from the Ministry of Education, Culture, Sports, Science, and Technology.^16^, ^17^ The cohort comprised 110,585 residents (46,395 men and 64,190 women, aged 40–79 years) from various districts in Japan who participated in our questionnaire survey during 1988–1990. The survey was conducted across 45 districts in 19 prefectures. The subjects of the present study were 26,464 men residing in the 24 districts wherein cancer incidence was reported. A follow‐up survey on the incidence and mortality rates in various cancers was conducted until the end of 2009. However, some study areas stopped the follow‐up survey of cancer incidence before 2009. Follow‐up was terminated in 1994, 1999, 2000, 2002, and 2003 in one study area each; it was terminated in 1997, 2006, and 2008 in two areas each.

We initially investigated survival rates using resident registration books in the municipalities for death due to prostate cancer, and the cause of death was confirmed from death certificates. We judged prostate cancer from the code C61 in the International Statistical Classification of Diseases and Related Health Problems, Tenth Revision.

The questionnaire covered medical history, family history, health status, health habits, dietary habits, favorites, alcohol consumption, smoking, occupation, height, body weight, residential area, education level, stress, marital status, and child‐bearing (delivery and pregnancy). Thirty‐two dietary items were covered in the questionnaire. Among dairy products, milk, cheese, butter, and yogurt were included. The frequency of eating these items was classified into five categories; “seldom,” “once or twice a month,” “once or twice a week,” “three or four times a week,” and “almost every day.” We reclassified these five categories into three groups (low, intermediate, and high consumption), calculated the risks of intermediate and high consumption, and compared the data with those of low consumption. In milk and yogurt, “seldom to twice a week” was classified as low, “three to four times a week” was classified as intermediate, and “almost every day” was classified as high consumption. In cheese and butter, “seldom” was classified as low, “once a month to two times a week” was classified as intermediate, and “over three times a week” was classified as high consumption. The validity of the questionnaire on dietary habits has been previously reported.^18^ The body mass index (BMI) was calculated from height and body weight and was classified into three categories: under 18.5 kg/m^2^, 18.6–25 kg/m^2^, and over 25.1 kg/m^2^. Total energy intake and its quartiles were calculated from the food frequency questionnaire.^19^

We analyzed hazard ratios (HRs) and 95% confidence intervals (CIs) related to age, family history of prostate cancer, and dietary intake using Cox's proportional hazard model, stratified by survey area. Regarding dairy products, the HRs of intermediate and high consumption were calculated in comparison to those of low consumption, adjusted according to age and family history of prostate cancer, stratified by survey area. BMI, total energy intake, and education were optionally added to the adjustment factors. The dose–response relationship of each HR (*p* for trend) was calculated by a linear function using quintile numbers. The PHREG procedure in the Statistical Analysis System (SAS) package was used for statistical calculations.

## 3 RESULTS

Table 1 shows age, BMI, family history of prostate cancer, and total energy intake of the subjects according to the frequency of dairy product intake. Although family history of prostate cancer showed no association with consumption of any dairy product, BMI and total energy intake showed a positive association with consumption of all dairy products.

**TABLE 1:** Characteristics of the subjects in the baseline survey (1988–1990) of the Japan Collaborative Cohort Study according to the frequency of dairy product consumption

Milk									
		Low level^a^		Middle level^b^		High level^c^		Total	*p* ^h^
		No.	%	No.	%	No.	%		
Age (years)		mean	SD^g^	mean	SD	mean	SD		
	Mean±SD^g^	56.7	10.2	56.2	10.3	58.8	10.2		
Family history of prostate cancer									
	No	9940	42.5	2949	12.6	10489	44.9	23378	0.164
	Yes	28	41.2	4	5.9	36	52.9	68	
	Unknown							2874	
Body mass index (kg/m^2^)									
	< 18.5	566	44.4	124	9.7	584	45.8	1274	< 0.05
	18.5–24.9	7457	42.3	2250	12.8	7938	45.0	17645	
	≥ 25.0	1925	45.3	540	12.7	1782	42.0	4247	
	Unknown							3154	
Total energy intake (kcal)									
	393–1380	2123	51.5	442	10.7	1555	37.7	4120	< 0.05
	1381–1685	1805	43.7	509	12.3	1819	44.0	4133	
	1686–2026	1721	41.5	534	12.9	1891	45.6	4146	
	2027–4262	1651	39.9	605	14.6	1878	45.4	4134	
	Unknown							9787	

Yogurt									
		Low level^a^		Middle level^b^		High level^c^		Total	*p*
		No.	%	No.	%	No.	%		
Age (years)		mean	SD	mean	SD	mean	SD		
	Mean±SD	56.5	10.1	58.4	10.5	60.1	10.1		
Family history of prostate cancer									
	No	17934	91.1	772	3.9	982	5.0	19688	0.570
	Yes	58	87.9	3	4.5	5	7.6	66	
	Unknown							6566	
Body mass index (kg/m^2^)									
	< 18.5	959	88.8	38	3.5	83	7.7	1080	< 0.05
	18.5–24.9	13777	91.3	592	3.9	721	4.8	15090	
	≥ 25.0	3266	91.8	134	3.8	159	4.5	3559	
	Unknown							6591	
Total energy intake (kcal)									
	393–1380	3635	93.0	120	3.1	152	3.9	3907	< 0.05
	1381–1685	3541	92.1	128	3.3	176	4.6	3845	
	1686–2026	3561	92.5	120	3.1	169	4.4	3850	
	2027–4262	3442	90.9	167	4.4	176	4.6	3785	
	Unknown							10933	

Cheese									
		Low level^d^		Middle level^e^		High level^f^		Total	*p*
		No.	%	No.	%	No.	%		
Age (years)		Mean	SD	Mean	SD	Mean	SD		
	Mean±SD	57.6	10.1	55.3	10.1	58.6	10.0		
Family history of prostate cancer									
	No	10078	49.6	8789	43.2	1470	7.2	20337	0.499
	Yes	31	44.3	36	51.4	3	4.3	70	
	Unknown							5913	
Body mass index (kg/m^2^)									
	< 18.5	531	51.6	406	39.5	92	8.9	1029	< 0.05
	18.5–24.9	7321	49.1	6495	43.6	1085	7.3	14901	
	≥ 25.0	1817	49.5	1627	44.3	229	6.2	3673	
	Unknown							6717	
Total energy intake (kcal)									
	393–1380	2583	63.2	1362	33.3	144	3.5	4089	< 0.05
	1381–1685	2075	50.5	1819	44.3	213	5.2	4107	
	1686–2026	1963	47.8	1863	45.4	281	6.8	4107	
	2027–4262	1743	42.3	1963	47.7	410	10.0	4116	
	Unknown							9901	

Butter									
		Low level^d^		Middle level^e^		High level^f^		Total	*p*
		No.	%	No.	%	No.	%		
Age (years)		Mean	SD	Mean	SD	Mean	SD		
	Mean± SD	57.2	10.1	55.4	10.1	58.7	10.6		
Family history of prostate cancer									
	No	10484	52.2	7892	39.3	1718	8.5	20094	0.488
	Yes	32	47.8	31	46.3	4	6.0	67	
	Unknown							6159	
Body mass index (kg/m^2^)									
	< 18.5	523	51.7	362	35.8	127	12.5	1012	< 0.05
	18.5–24.9	7649	52.0	5836	39.6	1238	8.4	14723	
	≥ 25.0	1890	51.9	1465	40.2	289	7.9	3644	
	Unknown							6941	
Total energy intake (kcal)									
	393–1380	2566	63.5	1268	31.4	206	5.1	4040	< 0.05
	1381–1685	2220	54.5	1578	38.7	275	6.8	4073	
	1686–2026	2112	52.0	1629	40.1	320	7.9	4061	
	2027‐4262	1825	44.6	1809	44.2	460	11.2	4094	
	Unknown							10052	

^a^ Seldom to twice/week.

^b^ Three to four times/week.

^c^ Almost everyday.

^d^ Seldom.

^e^ Once/month to twice/week.

^f^ Three times/week.

^g^ Standard diviation.

^h^ Peason's chi‐square test.

During the 697,777 person‐years of follow‐up, there were 412 cases of prostate cancer. The risk of prostate cancer increased with age, with an HR of 1.10 per 1‐year increase (95% CI: 1.09, 1.11). A family history of prostate cancer tended to be a risk factor, with an age‐adjusted HR of 3.90 (95% CI: 1.45, 10.47). Medians of time to diagnosis were 16.5, 16.7, 16.3, 14.3, and 9.8 years for the age at the time of recruitment of under 45 years, 45–49 years, 50–54 years, 55–59 years, and ≥60 years, respectively.

Milk consumption showed a positive association with the risk of developing prostate cancer adjusted for age and family history of prostate cancer, stratified by area. The HR for high consumption was 1.37 (95% CI: 1.08, 1.73), and a dose–response relationship was detected (*p* for trend = 0.009). Yogurt and cheese consumption showed positive correlations or dose–response relationships with prostate cancer, without any statistically significant difference (Table 2). Butter consumption alone showed a significant dose–response relationship (*p* for trend = 0.048).

**TABLE 2:** Hazard ratios (HRs) of the incidence of prostate cancer with 95% confidence intervals (CIs) for the consumption of dairy products

	Person ‐years	No. of cases	HR1^a^	95% CI		*p*	*p* for trend	HR2^b^	95% CI		*p*	*p* for trend	HR3^c^	95% CI		*p*		*p* for trend	HR4^d^	95% CI		*p*	*p* for trend
Milk (*n* = 24,220)																							
Low level^e^	165,980	126	Ref.					Ref.					Ref.					Ref.					
Middle level^f^	50,275	50	1.29	0.93	1.80	0.127	0.009**	1.32	0.94	1.85	0.114	0.011**	1.28	0.84	1.95	0.248		0.008**	1.38	0.85	2.24	0.196	0.039**
High level^g^	170,219	196	1.37	1.08	1.73	0.009**		1.37	1.08	1.74	0.010**		1.48	1.11	1.97	0.008	**		1.43	1.02	1.99	0.036**	
Yogurt (*n* = 20,518)																							
Low level^e^	302,638	222	Ref.					Ref.					Ref.						Ref.				
Middle level^f^	12,498	16	1.45	0.87	2.41	0.153	0.092*	1.47	0.87	2.49	0.150	0.151	1.22	0.60	2.48	0.591		0.041**	1.11	0.51	2.38	0.784	0.208
High level^g^	15,251	22	1.35	0.87	2.11	0.184		1.28	0.81	2.04	0.292		1.69	1.02	2.80	0.043	**		1.43	0.82	2.49	0.206	
Cheese (*n* = 20,407)																							
Low level^h^	160,112	137	Ref.					Ref.					Ref.						Ref.				
Middle level^i^	146,927	146	1.23	0.97	1.56	0.090*	0.259	1.26	0.99	1.60	0.065*	0.327	1.20	0.91	1.57	0.196		0.851	1.21	0.88	1.65	0.243	0.462
High level^j^	23,710	26	1.04	0.68	1.58	0.874		0.99	0.63	1.55	0.964		0.87	0.50	1.50	0.612			1.03	0.57	1.86	0.929	
Butter (*n* = 20,161)																							
Low level^h^	254,905	223	Ref.					Ref.					Ref.						Ref.				
Middle level^i^	46,085	53	1.26	0.94	1.71	0.128	0.048**	1.29	0.95	1.75	0.099*	0.036**	1.19	0.83	1.73	0.346		0.263	1.22	0.80	1.87	0.358	0.587
High level^j^	25,950	34	1.34	0.93	1.93	0.114		1.37	0.95	1.98	0.096*		1.22	0.77	1.93	0.401			1.05	0.60	1.83	0.874	

^a^ Adjusted for age and family history of prostate cancer (FHPCa), and stratified by area.

^b^ Adjusted for age, FHPCa and body mass index (BMI), and stratified by area.

^c^ Adjusted for age, FHPCa, BMI and total energy intake, and stratified by area.

^d^ Adjusted for age, FHPCa, BMI, total energy intake and education level, and stratified by area.

^e^ Seldom to twice/week.

^f^ Three to four times/week.

^g^ Almost everyday.

^h^ Seldom.

^i^ Once/ month to twice/week.

^j^ Three times/week.

* *p* < 0.1

** *p* < 0.05.

After adjusting for BMI, milk consumption continued to show a significant positive risk (HR = 1.32 for intermediate consumption and 1.37 for high consumption) and a dose–response relationship (*p* for trend = 0.011). Butter consumption was also associated with a tendency for positive risk and showed a significant dose–response relationship (*p* for trend = 0.048).

After the addition of both BMI and total energy intake to the adjustment factors, the consumption of milk and yogurt was still associated with a significant positive risk. The HR of high milk consumption was 1.48 (95% CI: 1.11, 1.97) and that of high yogurt consumption was 1.68 (95% CI: 1.02, 2.80). Both milk and yogurt showed a significant dose–response relationship (*p* for trend: milk, 0.008; yogurt, 0.041).

When the HR was additionally adjusted for education, the HR for high milk consumption (1.43, *p *= 0.036) and the dose–response (*p *= 0.039) were still significant. However, the HR for high yogurt consumption and the dose–response became nonsignificant. As Westernized lifestyle such as high intake of dairy products is thought to be associated with high education level in Japan, this adjustment may underestimate the risk of dairy products.

## 4 DISCUSSION

In Japan, only a few epidemiological studies have investigated the incidence of prostate cancer,^1^, ^2^, ^3^, ^4^, ^5^, ^6^, ^7^ and Hirayama conducted a cohort study a long time ago.^1^ With regard to dairy products, that study showed that high milk intake did not increase the risk of prostate cancer. However, some case–control studies reported that consumption of dairy products, especially milk, is a positive risk factor for the development of prostate cancer.^1^, ^2^

In the last 10 years, many epidemiological studies have been conducted,^5^, ^6^, ^7^, ^20^, ^21^, ^22^, ^23^, ^24^, ^25^, ^26^, ^27^, ^28^, ^29^, ^30^, ^31^, ^32^ including ours’.^29^, ^30^, ^31^, ^32^ Dietary habits,^6^, ^7^, ^23^, ^24^, ^25^, ^26^, ^27^, ^28^, ^32^ especially the intake of dairy products,^6^ were investigated. A case–control study that used a semi‐quantitative food frequency questionnaire reported a positive but non‐significant risk with milk consumption.^5^ The Japan Public Health Center–Based Prospective Study showed significant positive risk and dose‐response relationships for the consumption of dairy products.^6^

In the present study, the consumption of milk and yogurt was a risk factor for prostate cancer, and milk intake showed a significant dose–response relationship. While cheese and butter consumption showed positive correlations, statistical analyses deemed these relationships not to be significant.

Concerning the association between dairy product intake and prostate cancer development, contrasting results have been reported in literature. A recent meta‐analysis showed that all relative risks (RRs) of high consumption and dose–response for total prostate cancer ranged from 1.68 to 1.09 (1.07 per 400 g/d) for total dairy products. For milk (whole, low‐fat, and skim milk considered separately), the RRs ranged from 1.50 to 0.92 (95% CI: 1.06, 0.98 per 200 g/d), and for cheese, the RRs ranged from 1.18 to 0.74 (1.10 per 50 g/d).^11^ Another study reported that the RR of increasing risk of total prostate cancer for the intake of total dairy products was 1.07 (95% CI: 1.02, 1.12) per 400 g/d; the RR for total milk intake was 1.03 (95% CI: 1.00, 1.07) per 200 g/d, and the RR for low‐fat milk intake was 1.06 (95% CI: 1.01, 1.11) per 200 g/d; the RR for cheese intake was 1.09 (95% CI: 1.02, 1.18) per 50 g/d, and the RR for dietary calcium was 1.05 (95% CI: 1.02, 1.09) per 400 mg/d.^12^

Several studies tried to examine which components of dairy products may be associated with prostate cancer. A high fat diet was reported to be possibly associated with prostate cancer development.^33^ They are classified as animal/vegetable fat or saturated/unsaturated fatty acids in several ways. The possible risks of the development and progression of prostate cancer after the consumption of total and specific types of fat were investigated.^33^, ^34^ Dairy products contain large quantities of saturated fatty acids. Some ecological studies reported a close relationship between prostate cancer‐related death and fat and calorie intake. In other studies, although a relationship between fat intake and prostate cancer incidence was found, the cancer risk disappeared after adjustment for total energy intake.^33^ A meta‐analysis of the relationship between fatty acid intake and prostate cancer development showed that the evidence was limited, and no definite relationship could be reached between the consumption of total fat, saturated fatty acids, monounsaturated fatty acids, or polyunsaturated fatty acids and overall prostate cancer development and also “advanced/high‐grade” prostate cancer development.^35^ However, recent studies reported that each kind of fatty acids may have heterogeneous effects,^36^, ^37^, ^38^ which might explain the contradictory results of the role of dietary fat regarding prostate cancer development.^38^ In our results, the HR of milk intake was slightly decreased after adjusting for BMI. The risk associated with the intake of dairy products may thus be influenced by total energy intake.

Calcium and vitamin D in dairy products are suggested to play important roles in the pathogenesis of prostate cancer. It is hypothesized that the intake of high amounts of calcium inhibits the synthesis of 1,25(OH)2 vitamin D, thus increasing the risk of developing prostate cancer.^1^ Epidemiologically, the Health Professional Follow‐up Study reported that calcium was a risk factor for prostate cancer incidence independent of fat intake, especially for advanced and metastatic cancer (RR = 1.6 in advanced cases, RR = 1.8 in metastatic cases).^10^ In the Cancer Prevention Study II Nutrition Cohort, total calcium intake including supplements other than the intake through diet was examined, and the relationship with prostate cancer was investigated. However, another study similarly examining calcium intake concluded that moderate intake of calcium did not markedly increase prostate cancer risk.^39^ A positive relationship of total calcium and dairy calcium intakes, but not nondairy calcium or supplemental calcium intakes, with total prostate cancer risk was reported. Additional intake of calcium from food supplements was associated with an increased risk of fatal prostate cancer.^12^ In Japan, as in the Western world, milk and dairy products are major dietary sources of calcium intake. Calcium and vitamin D, in concurrence with saturated fat in dairy products, favor the development of prostate cancer.

Insulin‐like growth factor‐1 (IGF‐1) was suggested to be positively associated with the risk of prostate cancer in meta‐analyses.^40^, ^41^, ^42^ High‐energy intake^43^ and milk consumption^44^ may increase plasma IGF‐1 levels. One study suggested a link between fat intake and prostate cancer involving IGF‐1, insulin, or leptin.^45^ Moreover, another study showed that vitamin D levels increased circulating IGF‐1 levels.^46^

In our previous study, unfortunately, we could not show a correlation between serum IGF‐1 levels and prostate cancer incidence.^31^ The largest pooled analysis, including our previous study, investigating the association between circulating concentrations of IGFs (IGF‐I, IGF‐II, IGFBP‐1, IGFBP‐2, and IGFBP‐3) and prostate cancer risk, provided strong evidence that IGF‐I is highly likely to be involved in prostate cancer development.^47^

This study has some limitations. A drastic change in the methods to diagnose prostate cancer has occurred in the last 30 years. Prostate‐specific antigen (PSA) has been used in clinical practice since 1987, just before our baseline survey (1988–1990). The importance of PSA as a screening test has increased in the last decade worldwide, including Japan. Recently, many patients without any lower urinary tract symptoms were diagnosed with prostate cancer based on high PSA levels. Some municipalities in Japan performed mass screening for prostate cancer based on PSA levels, but no area surveyed in our study was included.

Moreover, our study did not collect detailed clinical information on cancer cases, such as information on serum PSA levels, tumor‐lymph node‐metastasis (TNM) stage, or pathological grade, because we obtained information about cancer incidence not from local hospitals but from local cancer registries. Thus, we could not investigate the risk adjusted for the characteristics of prostate cancer. The participants who visited a hospital regularly for some chronic disease might have had a higher probability of undergoing PSA testing. In our study, the opportunities to take a PSA test were not recorded. We tried to assess the risk of dairy products adjusted for diseases (diabetes mellitus, hypertension, and gastric ulcer) under treatment at the time of the baseline survey, but the results did not change (data not shown).

In summary, despite such limitations, our cohort study suggests that the intake of dairy products is an important risk factor for prostate cancer development in Japan. In particular, the data may provide further clues regarding the effects of high intake of fat, calcium, and IGFs. Further studies are needed to clarify which components of dairy products contribute to this increased risk. The consumption of dairy products is worthy of consideration when comparing Japanese and Western diets regarding the risk of developing prostate cancer.

## CONFLICT OF INTEREST

All authors declared no conflicts of interest on this study.

## AUTHORS CONTRIBUTIONS

All authors contributed to the conceptualization and methodology of the present study and took part in data collection. Akiko Tamakoshi performed the administration of the whole project of the JACC Study. Data analysis was performed by Kazuya Mikami and Kotaro Ozasa. Tsuneharu Miki, Yoshiyuki Watanabe, Mitsuru Mori, Koji Suzuki, and Kenji Wakai supervised and provided advice from the viewpoint of their expertise in urology and epidemiology. The first draft of the manuscript was prepared by Kazuya Mikami, and all authors edited, reviewed, and approved it.

## ETHICAL APPROVAL STATEMENT

This study was approved by the ethics committees of Hokkaido University, Hokkaido, Japan, and Osaka University, Osaka, Japan. number/ID 14285‐6.

## Data Availability

The data analyzed in this study are available from the corresponding author upon reasonable request.
